# Follistatin-Like 3 Enhances Invasion and Metastasis via β-Catenin-Mediated EMT and Aerobic Glycolysis in Colorectal Cancer

**DOI:** 10.3389/fcell.2021.660159

**Published:** 2021-07-28

**Authors:** Yuqiang Li, Mengxiang Tian, Wenxue Liu, Dan Wang, Zhongyi Zhou, Qian Pei, Yan Huang, Fengbo Tan, Cenap Güngör

**Affiliations:** ^1^Department of General Surgery, Xiangya Hospital, Central South University, Changsha, China; ^2^National Clinical Research Center for Geriatric Disorders, Xiangya Hospital, Central South University, Changsha, China; ^3^Department of General, Visceral and Thoracic Surgery, University Medical Center Hamburg-Eppendorf, Hamburg, Germany; ^4^Department of Cardiology, Xiangya Hospital, Central South University, Changsha, China; ^5^Key Laboratory of Protein Chemistry and Developmental Biology of Ministry of Education, College of Life Sciences, Hunan Normal University, Changsha, China; ^6^Department of Neurosurgery, Second Affiliated Hospital of Hunan Normal University, Changsha, China; ^7^Hunan Provincial Key Laboratory of Neurorestoration, Changsha, China

**Keywords:** FSTL3, β-Catenin, colorectal cancer, EMT, aerobic glycolysis, metastasis

## Abstract

Previous studies reported that Follistatin-like 3 (FSTL3) is abundantly expressed in several solid tumors and participate in the regulation of cell metabolism. However, the clinico-pathological significance, biological role and molecular mechanism of FSTL3 in colorectal cancer (CRC) is still unclear. Here we report that the expression level of FSTL3 in colon cancer specimens was significantly higher, compared to normal tissue and interestingly, the expression of FSTL3 was related to lymph node metastasis, tumor stage, tumor size, and intravascular emboli (IVE). As an upstream molecular event, we found that transcriptional regulation of FSTL3 was highly dependent on YAP1 de-phosphorylation events and that increased FSTL3 expression readily activated the β-Catenin pathway, which is a well-known signaling hub that promotes EMT processes and aerobic glycolysis in cancer cells. We found that elevated FSTL3 expression strongly promotes migration, invasion and metastatic formation of CRC cells by directly activating β-Catenin -mediated EMT and aerobic glycolysis. In the xenograft mouse model, FSTL3 expression was linked to increased metastatic formation of CRC cells. Together, the activation of YAP1 induces FSTL3 expression. FSTL3-mediated β-Catenin pathway activation promotes EMT and aerobic glycolysis and therefore affecting the invasive and metastatic capacity of CRC cells. The abundant FSTL3 expression is a poor prognostic factor and pharmacological targeting of YAP1 can counteract FSTL3 expression, suggesting a promising therapeutic target for anti-metastatic strategies in patients suffering from CRC.

## Highlights

- The high expression of FSTL3 is a poor prognostic factor for patients with CRC.
- The activation of YAP1 can induce FSTL3 expression, which then promotes EMT and enhances aerobic glycolysis to affect invasion and metastasis formation of CRC cells by activating the β-Catenin pathway.
- This study provides a new crosstalk mechanism between Hippo/YAP and Wnt/β-Catenin pathways, which suggests a new strategy for colorectal cancer treatment.

## Introduction

Colorectal cancer (CRC) is the third most frequent cancer which results in the 2nd cancer-related mortality worldwide (Siegel et al., 2020). Treatment modalities for CRC includes surgical resection, chemotherapy, and/or radiation therapy. In recent years, the prognosis of patients with CRC has been significantly improved due to advances in surgery combined with adjuvant therapy in the past decades (Li Y. et al., 2019). However, invasion and metastatic formation of tumor cells are still the main causes of death in patients suffering from CRC (Siegel et al., 2020). Hence, the exploration of key molecules and their related molecular mechanisms regulating invasion and metastasis can provide prognostic markers and potentially new therapeutic targets for the treatment course of CRC in future settings.

Epithelial-mesenchymal transition (EMT) is a process in which cells gradually loses their epithelial phenotype and transforms into a mesenchymal phenotype. EM-transitioned cells are linked to: (i) enhanced cell motility, (ii) promotes transition from *carcinoma in situ* to an invasive carcinoma, (iii) enables tumor cells to invade into neighboring tissues (vascular/lymphatic) and promoting the metastatic cascade (Sánchez-Tilló et al., 2011). Increasing evidences demonstrate that EMT can also initiate the metastatic progression of CRC (Thiery et al., 2009; Sleeman and Thiery, 2011; Dongre and Weinberg, 2019). The molecular characteristics of EMT contain the suppression of epithelial markers (e.g., *E*-cadherin) and the concomitant promotion of mesenchymal markers such as *N*-cadherin, Fibronectin1, and Vimentin (Kalluri and Weinberg, 2009). Therefore, exploration of the role and molecular mechanism of key genes that regulate EMT can provide a basis for controlling the invasion and metastatic formation of CRC cells.

Moreover, cancer cells rewire their metabolism and breakdown glucose to lactate in the presence of oxygen (aerobic glycolysis), a phenomenon known as the Warburg effect (Vander Heiden et al., 2009; Lunt and Vander Heiden, 2011). The alteration of metabolism endues cancer cells the ability to survive and grow in a nutrient-deficient and highly hypoxic tumor microenvironment (Binnewies et al., 2018; Sullivan, 2018). This metabolic re-programming involves altered expression and post-translational modification of several key metabolic enzymes. However, the molecular mechanisms underlying the altered expression or post-translational modifications of key metabolic proteins in CRC tumors are still not fully understood. Much attention has been paid to the glucometabolic re-programming of tumor cells in recent years. Drugs targeting key regulators of aerobic glycolysis effectively inhibit tumor progression *in vitro* and *in vivo* (Ganapathy-Kanniappan and Geschwind, 2013). Therefore, it is still necessary to further investigate key genes of aerobic glycolysis in CRC.

Follistatin-like 3 (FSTL3) is expressed in various normal human tissues (Tsuchida et al., 2000). Increasing evidences indicated that FSTL3 plays an important role in regulating embryonic development, osteogenesis, glucose, and lipid metabolism (Mukherjee et al., 2007). Moreover, FSTL3 was recently found abundantly expressed in non-small cell lung cancer (Gao et al., 2020) and breast cancer (Couto et al., 2017), and participates in tumor progression including invasion and metastasis. FSTL3 is an independent risk factor and is linked with poor prognosis within various cancers. However, the molecular mechanisms and impact of FSTL3 on CRC progression is still unclear.

YAP1, a key molecule in the HIPPO pathway, can translocate into the nucleus upon dephosphorylation where it functions to regulate and maintain cancer stem cell properties as well as the invasion and metastatic ability of CRC cells (Tan et al., 2018). Meanwhile, β-Catenin, the rate-limiting molecule of Wnt pathway, is involved in the regulation of various physiological events in CRC cells. Recent studies indicated that the crosstalk between the HIPPO/YAP1 and Wnt/β-Catenin signaling pathways can play a key role in the progression of CRC (Konsavage et al., 2012; Jiao et al., 2017). Various clinical trials with HIPPO/YAP1-inhibitors or Wnt/β-Catenin-inhibitors have already been started in solid tumors^1^. However, therapeutical targets inhibiting the crosstalk between the two signal pathways still needs to be discovered.

Our study revealed that increased FSTL3 expression is a poor prognostic factor in CRC patients and that transcriptional activation of FSTL3 is strongly induced following YAP1 activation. Additionally, abundant FSTL3 expression promotes EMT and enhances aerobic glycolysis to positively affect the invasive and metastatic capacity of CRC cells by activating the β-Catenin pathway. Our findings illustrate that FSTL3 could serve as a bridging molecule in the crosstalk between HIPPO/YAP1 and Wnt/β-Catenin pathways and that FSTL3 is a crucial regulatory factor of the β-Catenin molecular mechanisms in CRC. Therefore, therapeutically targeting of either FSTL3 and/or YAP1 is may be a promising anti-metastatic strategy in CRC patients.

## Materials and Methods

### Patients and Specimens

Tumor and matched para-carcinoma tissues were removed by radical resection from 130 stage III CRC patients without preoperative chemotherapy or radiotherapy at the Xiangya Hospital of Central South University (Changsha, China) randomly. The samples were then embedded in paraffin. Follow-up of patients was terminated on September 1st, 2018. Disease-free survival (DFS) was defined as the time to any event, irrespective of cause, except for any second primary cancers. Recurrence of or death from the same cancer and all treatment-related deaths or deaths from other causes are events. Second primary same cancers and other primary cancers were ignored, and loss to follow-up is censored. Overall survival (OS) was defined as the time to death, irrespective of cause. Local recurrence, distant metastases, second primary CRCs, and second other primary cancers were ignored. Loss to follow-up is censored. All procedures were in compliance with the ethical guidelines of the Xiangya Hospital. The normal mucosa was excised 5cm away from the tumor and was confirmed by pathologists for absence of tumor cells. Tumor stage was classified according to the 7th edition of the AJCC TNM staging system for CRC.

### Cell Culture and Reagents

The CRC cell lines [HT-29 (RRID: CVCL_0320), SW480 (RRID: CVCL_0546), SW620 (RRID: CVCL_0547), LOVO (CVCL_0399), HCT116 (RRID: CVCL_0291), DLD1 (RRID: CVCL_0248), and RKO (RRID: CVCL_0504)] were purchased from American Type Culture Collection (ATCC, United States). The cell lines were incubated in a humidified atmosphere with 5% CO_2_ at 37°C and cultivated in the recommended growth medium, supplemented with 10% FBS, 100 mg/ml streptomycin and 100 U/mL penicillin (Sigma-Aldrich, United States). The YAP inhibitor, Verteporfin (VP) was purchased from Selleck Chemicals (Houston, TX, United States).

### Western Blotting (WB)

The WB assay was performed as previously described (Tan et al., 2015). CRC cells were homogenized and lysed in RIPA buffer supplemented with protease inhibitors. Equal amounts of proteins were loaded and separated on 6% SDS-PAGE gel. Following electrophoresis, proteins were transferred to a PVDF membrane (Millipore, United States), the membrane was blocked in 5% (w/v) non-fat milk and incubated with the primary antibodies overnight, and followed by secondary antibody incubation (1:2000 dilution, CST, United States) for 1 h. Bands were visualized and quantitated using the ECL Advance Detection System (Millipore, United States). The primary antibodies used for WB analysis are listed in Supplementary Table 1.

### Quantitative Real-Time Polymerase Chain Reaction (qRT-PCR)

The qRT-PCR assay was performed as previously described (Tan et al., 2015). Total RNA was extracted from cells and tissues using TRIzol Reagent (TAKARA, Japan), and equal amounts of RNA were used for real-time qRT-PCR analysis (TAKARA, Japan) according to the manufacturer’s instructions. GAPDH was used as loading control. Primers are listed in Supplementary Table 2. The mRNA expression was quantitated using the 2-(^△^^Ct^ sample–^△^^Ct^ control) method.

### Wound-Healing/Scratch Assay

The wound-healing assay was performed as previously described (Li C. et al., 2019). The transfected cells were cultured in 6-well plates. After the cells reached 90–95% confluence, a standard 200 μl pipette tip was subsequently utilized to scratch linear wounds. In addition, cell monolayers were cultivated in FBS-free medium. After scratching, the images of the wound closure were captured at 0 and 36 h.

### Transwell Migration and Invasion Assay

The Transwell migration and invasion assay was performed as previously described (Li C. et al., 2019). 8 × 10^4^ cells, suspended in medium without FBS, were seeded into Transwell chambers (Costar Corning, United States), with or without Matrigel (Sigma-Aldrich, United States) coating. The lower chamber contained medium with 10% FBS. After 24 h, the migratory/invasive cells on the lower surface of the chamber were photographed and counted in 10 randomly selected microscopic fields after crystal violet staining.

### Immunofluorescence (IF)

The IF assay was performed as previously described (Tan et al., 2015). Cells cultured in 24-well chamber slides were washed twice with cold PBS, fixed with 4% paraformaldehyde for 10 min, permeabilized with 0.1% Triton-X for 5 min, blocked with 5% BSA, and incubated with primary antibodies at 4°C overnight. The cells were then stained with secondary antibodies and DAPI (4,6-diamidino-2-phenylindole) to visualize the nuclei. Images were acquired using a confocal microscope (Leica, Germany). The primary antibodies used for IF analysis are listed in Supplementary Table 1.

### Immunohistochemistry (IHC)

The protocol for IHC staining was performed as previously published (Davidson et al., 2013). The IHC staining results were evaluated by two independent pathologists (double-blinded). Briefly, the percentage of stained tumor cells (0: 0–5%; 1: 6–25%; 2: 26–50%; 3: 50–100%) and staining intensity scores (0: negative; 1: weak; 2: moderate; 3: strong) were summed. The CRC tissues were categorized into four groups: negative: ≤5% cells stained, regardless of intensity; weak expression: 1–2 points; moderate expression: 3–4 points; and strong expression: 5–6 points. The total score ≥ 3 was classified as significant overexpression and was considered as positive expression. Antibodies used for the IHC analysis are listed in Supplementary Table 1.

### Transduction/Transfection

Different lentiviral vectors with FSTL3-shRNA and negative-control shRNA were purchased from GENECHEM (Shanghai, China). HCT-116 and DLD-1 cells were transduced with FSTL3-shRNA and control-shRNA. Scrambled control-siRNA (siScr) and si-YAP1 were purchased from RiboBio (Guangzhou, China). HCT-116 cells were transduced using lentiviral vectors with siRNA targeting YAP1. For YAP1 overexpression, DLD-1 cells were transduced using lentiviral vectors carrying either full-length YAP1 cDNA or control sequences, respectively (GENECHEM, Shanghai, China). DLD-1 cells were transduced with lentiviral vectors containing shRNA-sequences targeting β-Catenin or control-shRNA, which were purchased from GENECHEM (Shanghai, China). Efficient knockdown and overexpression were detected by WB and qRT-PCR analysis.

### Luciferase Reporter Assay

Luciferase reporter assay was performed in HCT-116 and DLD-1 cell lines stably harboring luciferase reporter plasmids fused to the promoter region of human FSTL3 (Supplementary Figure 1). Cells were collected and cell extracts were prepared. Luciferase activity was measured using Luciferase assay kit (Promega, China) according to the manufacturer’s instructions.

### CRC – Mice Model

Female BABL/c athymic nude mice purchased from Hunan SJA Laboratory Animal Co., Ltd (Changsha, Hunan, China). Aged 5–6 weeks were used. Ethical statement was approved by the Ethics Committee of XiangYa Hospital Central South University (Changsha, China). All animal care and procedures were performed according to the guidelines on treating experimental animals well formulated by the ministry of science and technology of China. The capacity to metastasize to the liver was determined following a previously described method (Gavert et al., 2007). Briefly, all mice were anesthetized by inhalation of isoflurane (0.5–1.0%) and oxygen. Through a 1-cm incision in the upper left lateral abdomen, the spleen was exposed and 10^6^ cells suspended in 20 μL phosphate-buffered saline (PBS) were injected into the distal tip of the spleen using a Hamilton syringe. Following injection, the spleen was replaced in the abdomen and the incision was closed with staples. The animals were sacrificed after 5–6 weeks and spleen and liver were isolated and paraffin embedded.

### ATP Assay/Metabolism

Intracellular ATP production was measured using an ATP Assay Kit (Abcam, United States). Lactate production were measured using L-Lactate Assay Kit (Abcam, United States) and glucose uptake were measured using a Glucose Uptake Assay Kit (Abnova, China) according to the manufacturers’ instructions, respectively.

### Statistical Analysis

The data analysis was conducted using a Student’s *t*-test for the comparison between groups. The χ^2^ test was utilized to evaluate the association between protein levels and clinical characteristics. The correlations in gene expression levels were analyzed by Spearman’s rank correlation coefficients. Differences were thought to be significant at ^∗^*p* < 0.05, ^∗∗^*p* < 0.01, ^∗∗∗^*p* < 0.001, and ^****^p < 0.0001. n.s, no significance. The results were analyzed with SPSS 19.0 software (SPSS Inc., United States). All *in vitro* experiments were repeated at least three times.

## Results

### FSTL3 Is Frequently Overexpressed in CRC Tissues

**FIGURE 1:**
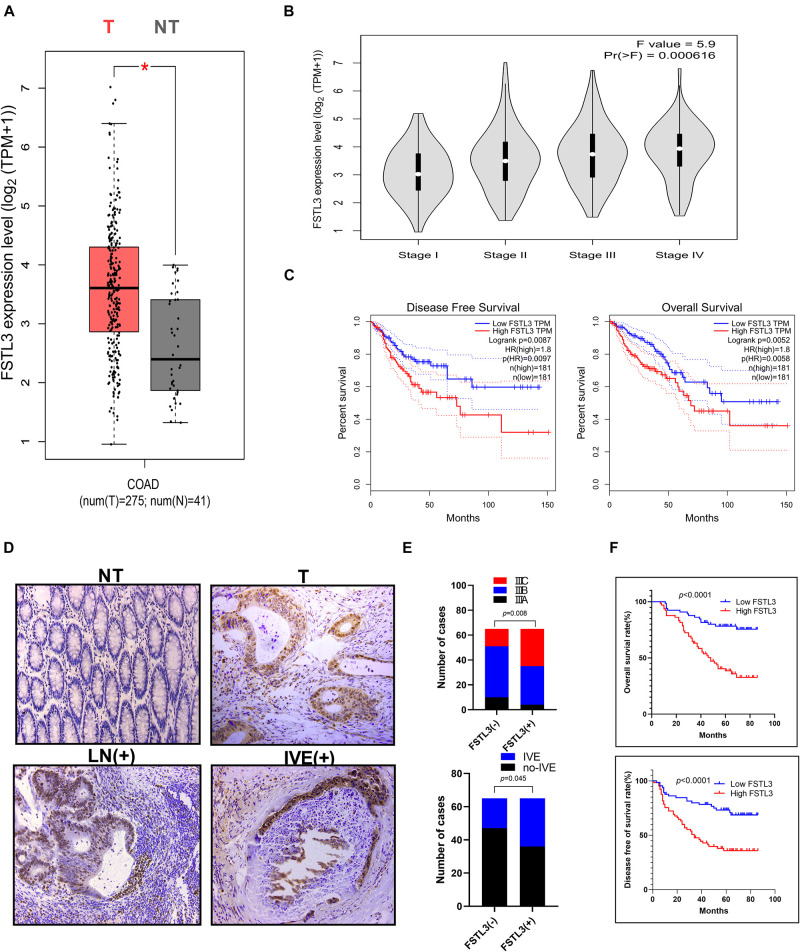
FSTL3 is in CRC. **(A)** The box plots indicated differential FSTL3 expression between CRC specimens (T) and paired normal tissues (NT), analyzed within the GEPIA database. *Differences were thought to be significant. **(B)** The pathological stage plot compared FSTL3 expression in different CRC stages, analyzed within the GEPIA database. **(C)** The survival plots demonstrated that FSTL3 is obviously related to DFS (*p* = 0.0087) and OS (*p* = 0.0052), analyzed within the GEPIA database. **(D)** Representative immunohistochemistry images of FSTL3 expression levels in NT, T, LN (metastatic lymph node tissue), and IVE (intravascular emboli). **(E)** The histogram indicates that FSTL3 expression correlates with tumor stage and IVE. **(F)** Survival plots were obtained to verify that FSTL3 expression is obviously related to DFS and OS (*p* < 0.0001).

The data from the GEPIA database^2^ illustrated that FSTL3 was significantly overexpressed in CRC specimens (T) relative to the adjacent normal colonic mucosa (NT) (Figure 1A). The mRNA expression levels of FSTL3 were correlated with the AJCC staging of CRC (Figure 1B). Moreover, the survival curves demonstrated that FSTL3 was obviously related to disease-free survival (DFS) (*p* = 0.0087) and overall survival (OS) (*p* = 0.0052) in GEPIA database (Figure 1C). In addition, the data from other databases, including the Human Protein Atlas project^3^, OncoLnc^4^, GSE17536 and GSE41258, further displayed that abundant FSTL3 expression was a poor prognostic factor in CRC patients (Supplementary Figure 2). Then, 130 CRC tumor and matched para-carcinoma tissues were randomly collected from the Xiangya Hospital of Central South University (Changsha, China). The correlation between FSTL3 expression and clinico-pathological characters of 130 CRC patients was summarized in Table 1. The expression of FSTL3 significantly correlated to lymph node metastasis, staging, tumor size and intravascular emboli (IVE). The immunohistochemistry images indicated low- or negative expression levels of FSTL3 in the paired normal tissues (NT) and high-expression in tumor (T), metastatic lymph node (LN), and IVE tissues (Figure 1D). Moreover, there were significant differences regarding sub-staging in stage III CRC (*p* = 0.045) and formation of IVE (*p* = 0.008) between the FSTL3 high and low expression groups (Figure 1E). Our data also confirmed that expression of FSTL3 was obviously related to DFS (*p* < 0.0001) and OS (*p* < 0.0001) (Figure 1F and Table 2). Collectively, our data suggest that elevated FSTL3 expression is associated with a worse outcome in CRC patients.

**TABLE 1:** Correlation between FSTL3 expression and clinicopathological characters of 130 CRC patients.

**Clinicopathological characters**			**FSTL3 expression**			
		***n***	**Low (*n* = 65)**	**High (*n* = 65)**	***p-*value**
Age(years)	<60	80	42	38	0.471
	≥60	50	23	27	
Gender	Female	75	26	29	0.594
	Male	55	39	36	
Tumor location	LCC	28	17	11	0.369
	RCC	29	15	14	
	REC	73	33	40	
Tumor differentiation	G1	91	48	43	0.265
	G2	23	8	15	
	G3	16	9	7	
Depth of invasion	T1–T2	16	11	5	0.109
	T3–T4	114	54	60	
Lymph node metastasis	N1	80	50	30	0.000*
	N2	50	15	35	
Stage of III	IIIA	14	10	4	0.008*
	IIIB	72	41	31	
	IIIC	44	14	30	
CEA (ng/ml)	<5.0	98	48	50	0.684
	≥5.0	32	17	15	
Diameter(cm)	<5.0	76	45	31	0.013*
	≥5.0	54	20	34	
Intravascular emboli	No	83	47	36	0.045*
	Yes	47	18	29	
Intestinal obstruction	No	119	59	60	0.753
	Yes	11	6	5	
CA19-9(kU/L)	<35.0	109	54	55	0.812
	≥35.0	21	11	10	

*p < 0.05 was considered statistically significant. The *P*-values of chi-square test.

**TABLE 2:** Univariate and multivariate analysis of 130 CRC patients for DFS and OS.

**Variables**	**DFS**				**OS**			
	**Univariate analysis**		**Multivariate analysis**		**Univariate analysis**		**Multivariate analysis**	
	**HR(95%CI)**	***p*-value**	**HR(95%CI)**	***p-*value**	**HR(95%CI)**	***p-*value**	**HR(95%CI)**	***p*-value**
Age, years (≥60 vs. <60)	1.080(0.664–1.811)	0.771	NA	NA	1.025(0.599-1.753)	0.930	NA	NA
Gender (male vs. female)	1.076(0.642-1.804)	0.780	NA	NA	1.134(0.663-1.938)	0.646	NA	NA
Tumor location		0.740	NA	NA		0.836	NA	NA
(LCC vs. RCC)	0.757(0.354-1.617)	0.472	NA	NA	0.782(0.350–1.747)	0.550	NA	NA
(LCC vs. REC) (RCC vs. REC)	0.819(0.469–1.508) 0.924(0.477–1.790)	0.521 0.815	NA NA	NA NA	0.893(0.469–1.702) 0.876(0.442–1.738)	0.731 0.705	NA	NA
Type G1 vs. G3 G2 vs. G3 G1 vs. G2	0.600(0.289–1.246) 1.165(0.504–2.694) 1.941(1.047–3.597)	0.072 0.171 0.720 0.035	0.836(0.382–1.830) 0.901(0.357–2.274) 1.077(0.533–2.177)	0.899 0.654 0.825 0.835	0.528(0.252–1.105) 1.151(0.498–2.660) 2.181(1.165–4.083)	0.028 0.090 0.742 0.015	0.794(0.356–1.771) 0.797(0.313–2.030) 1.004(0.496–2.033)	0.846 0.572 0.634 0.991
Depth of invasion(T3/T4 vs. T1/T2) Substage IIIA vs. IIIB IIIA vs. IIIC IIIC vs. IIIB Diameter(≥5 cm vs. <5 cm)	0.901(0.428–1.898) 0.908(0.376–2.195) 1.650(0.679–4.011) 0.550(0.322–0.940) 1.822(1.097–3.024)	0.784 0.083 0.831 0.269 0.029 0.020	NA NA 1.298(0.517-3.255) 0.487(0.096-2.464) 0.632(0.161-2.480) 1.822(1.064-3.119)	NA 0.681 0.579 0.384 0.511 0.029	0.987(0.447-2.180) 0.981(0.375-2.563) 2.135(0.819-5.565) 0.459(0.265-0.796) 1.740(1.029-2.940)	0.975 0.016 0.969 0.121 0.006 0.039	NA 1.345(0.497-3.640) 1.952(0.386-9.883) 0.689(0.182-2.604) 1.729(0,989-3.020)	NA 0.714 0.560 0.419 0.583 0.055
LN metastasis(N1 vs. N2) Intravascular emboli Intestinal obstruction	1.658(0.997–2.756) 3.876(2.302–6.527) 1.025(0.410–2.562)	0.051 0.000 0.958	0.645(0.169–2.465) 3.163(1.721–5.815) NA	0.522 0.000 NA	2.113(1.250–3.574) 4.608(2.660–7.982) 1.014(0.405–2.543)	0.005 0.000 0.976	0.892(0.242–3.286) 3.455(1.852–6.445) NA	0.864 0.000 NA
CEA(≥ 5 ng/ml vs. < 5ng/ml)	0.964(0.530–1.753)	0.903	NA	NA	1.122(0.612–2.055)	0.709	NA	NA
CA199(≥35 kU/L vs. <35 kU/L) FSTL3 expression level (High vs. Low)	1.800(0.973–3.331) 2.866(1.659–4.953)	0.061 0.000	NA 1.948(1.059–3.586)	NA 0.032	1.617(0.854–3.063) 3.674(2.027–6.660)	0.140 0.000	NA 2.424(1.279–4.593)	NA 0.007

*HR, hazard ratio; CI, confidence interval; NA, not available.*

### FSTL3 Promotes Migration and Invasion of CRC Cells *in vitro*

**FIGURE 2:**
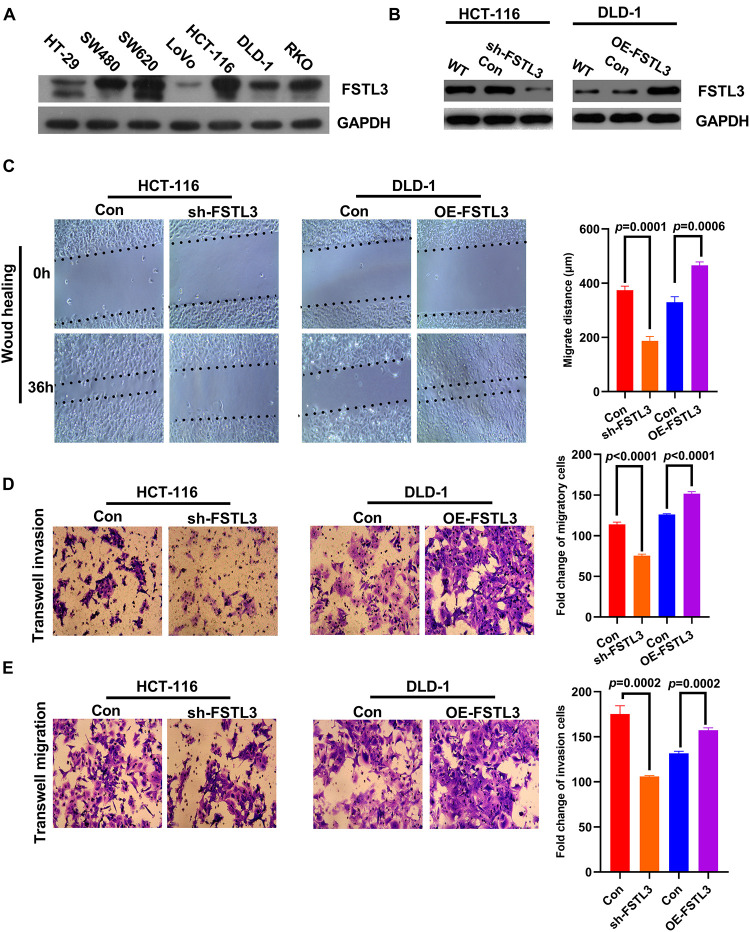
FSTL3 promotes migration and invasion of CRC cells *in vitro*. **(A)** Protein expression levels of FSTL3 in CRC cell lines. **(B)** WB analysis of FSTL3 knockdown and overexpression. **(C)** The wound-healing assays were applied to detect migration capacity (timepoints 0 and 36 h) affected by FSTL3 in HCT-116 and DLD-1 cells. **(D,E)** Transwell assays were utilized to quantify migration and invasion capacity affected by FSTL3 in HCT-116 and DLD-1 cells. Data are presented as the mean ± SD from three independent experiments.

FSTL3 protein expression levels were investigated in CRC cell lines and SW480, SW620, HCT-116, and RKO exhibited high-expression of FSTL3, whereas HT-29, Lovo and DLD-1 cells showed low-expression of FSTL3 (Figure 2A). HCT-116 and DLD-1 cell lines were selected for further investigations because of their invasive and migrative capabilities. Stable FSTL3-knockdown (HCT-116) and FSTL3-overexpression (DLD-1) cell lines were then established to examine the molecular function of FSTL3 (Figure 2B). The effect of FSTL3 on migration capacity in CRC cells was detected using the wound-healing/scratch and transwell assays. These results demonstrated that CRC cells with elevated FSTL3 expression have enhanced migrative ability, compared to their respective control cells (Figure 2C). One disadvantage of the scratch assay is, that it is not quantitative. Therefore, we also performed transwell assays (with and without matrigel) and confirmed that elevated FSTL3 expression was able to strengthen the migrative and invasive capacity of CRC cells *in vitro* (Figures 2D,E). In sum, elevated FSTL3 expression promoted migration and invasion of CRC cells *in vitro*.

### FSTL3 Expression Is Regulated by YAP1 in CRC Cells

**FIGURE 3:**
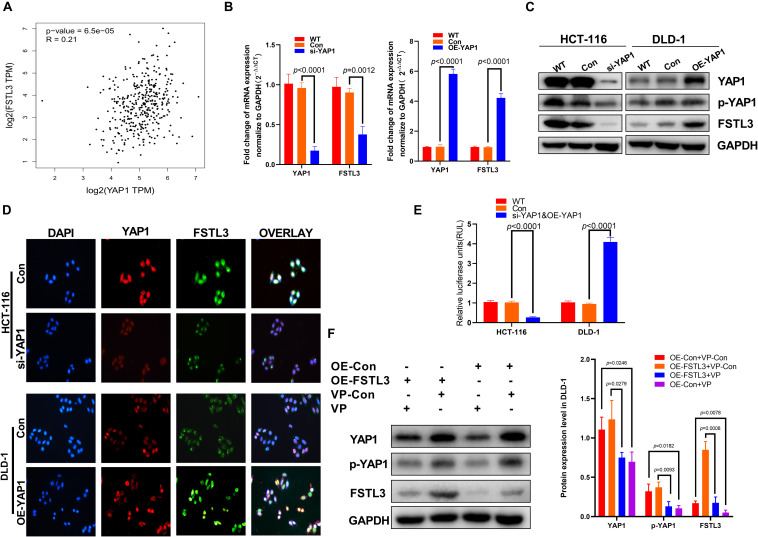
YAP1 modulates the expression of FSTL3 in CRC. **(A)** Data derived from the GEPIA database showed positive correlation between FSTL3 and YAP1 in CRC tissues. **(B)** (left) YAP1 knockdown (siRNA) decreased FSTL3 mRNA expression in HCT116 cells, quantified with qRT-PCR; (right) YAP1 mRNA overexpression in DLD-1 cells promotes FSTL3 expression, quantified with qRT-PCR. **(C)** WB analysis of si-YAP1 and OE-YAP1 in HCT-116 and DLD-1 cells. **(D)** Immunofluorescence displayed elevated FSTL3 expression following YAP1 overexpression in DLD-1 cells. **(E)** Luciferase assays for the *hFSTL3* gene promoter construct. Reporter activation was analyzed in si-YAP1 and OE-YAP1 CRC cells. **(F)** WB analysis determined reduced YAP1 phosphorylation and FSTL3 expression levels in Verteporfin (VP)-treated DLD-1 cells.

As a transcriptional cofactor, Yes-associated protein-1 (YAP1) is known to modulate the transcriptional activity of various transcription factors to regulate and maintain cancer stem cell properties and organ size during embryonic development (Tan et al., 2018). A recent study indicated that the mRNA expression of YAP1 is correlated with that of FSTL3 (Lee et al., 2017). We therefore hypothesized that YAP1 is able to modulate FSTL3 gene expression in CRC cells. Initially, we found a significant correlation between FSTL3 and YAP1 expression in CRC using the GEPIA database (see footnote 2) (Figure 3A). To pinpoint whether YAP1 is responsible for increased FSTL3 gene expression, we established YAP1-knockdown as well as YAP1-overexpression cell clones. According to baseline FSTL3 expression levels (Figure 2B), we decided to knockout YAP1 expression in HCT116 cells and to overexpress YAP1 in DLD-1 cells. Effective YAP1 knockout and overexpression was confirmed by qRT-PCR, WB, and immunofluorescence, respectively (Figures 3B–D). We found substantially decreased FSTL3 mRNA and protein expression in YAP1 knockout HCT116 cells, whereas YAP1-overexpression in DLD-1 cells displayed increased FSTL3 mRNA and protein levels (Figures 3B–D). *In silico* analyses of the human *FSTL3* (*hFSTL3*) gene promoter sequence revealed three potential YAP1 binding sites, upstream to the transcriptional start site of the *hFSTL3* (Supplementary Figure 1). Next, we investigated whether YAP1 directly interacts with the *hFSTL3* gene promoter using luciferase reporter-gene assays. We therefore cloned the promoter region of *hFSTL3* into GV238 vector and measured luciferase expression in YAP1-knockout HCT-116 and YAP1-overexpressed DLD-1 cells. We detected decreased luciferase expression in YAP1-knockout HCT-116 cells and increased luciferase expression in YAP1-overexpressed DLD-1 cells (Figure 3E). For proof-of-principle, we decided to inhibit YAP1 activity by treating FSTL3-overexpressing DLD-1 and DLD-1-vector control cells with 0.5 μM Verteporfin (VP) for 48 h. The treatment with VP showed substantially reduced FSTL3 expression levels in DLD-1-OE-FSTL3 and DLD-1-vector control cells (Figure 3F). Collectively, these data suggest that YAP1 is indeed necessary for transactivation of FSTL3 in CRC cells.

### FSTL3 Modulates β-Catenin-Mediated EMT Processes in CRC Cells

**FIGURE 4:**
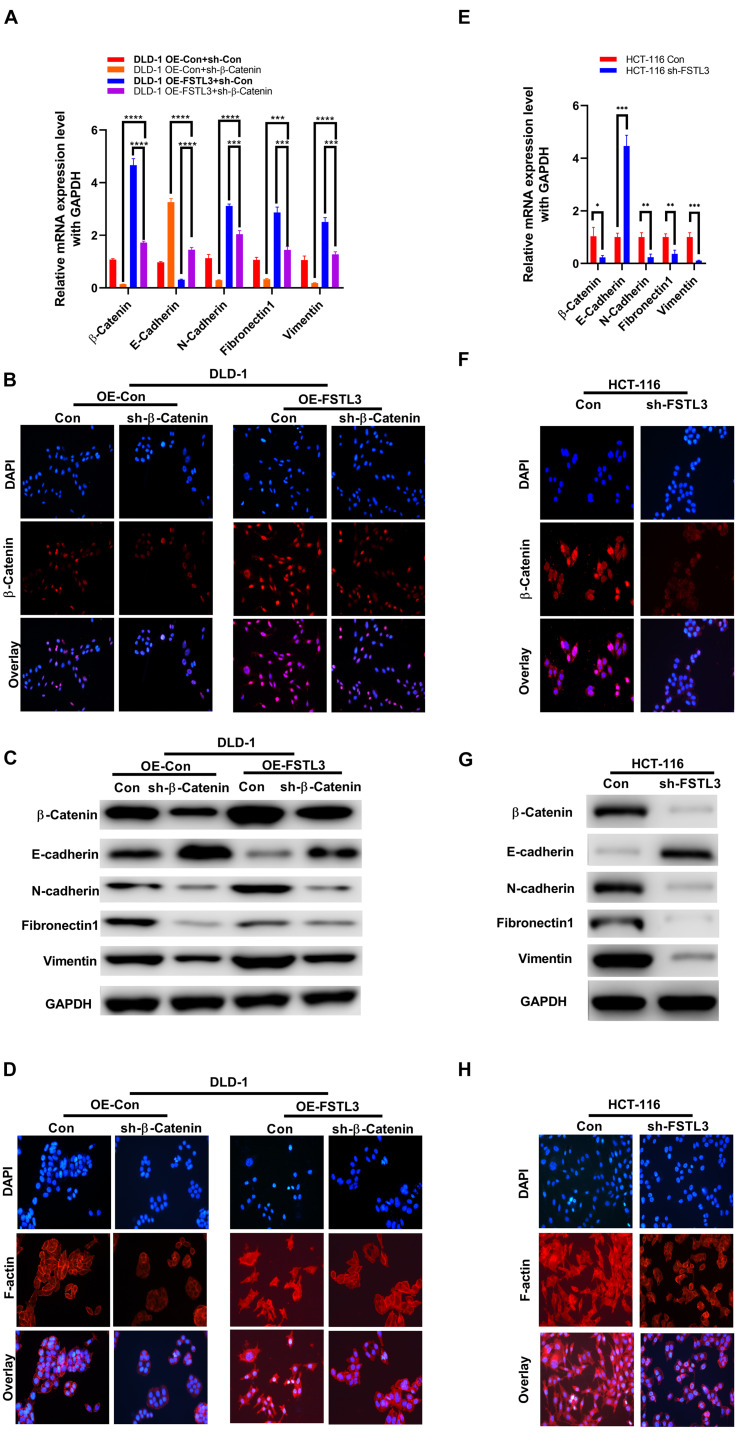
FSTL3 induces β-Catenin-mediated EMT. **(A)** qRT-PCR displayed elevated β-Catenin mRNA expression in DLD-1 cells co-expressed with FSTL3. Impact of FSTL3 and β-Catenin expression on EMT in DLD-1 cells. ****p < 0.0001; ***p < 0.001. **(B)** Immunofluorescence of ectopically overexpressed FSTL3 promoted expression and nuclear translocation of β-Catenin, compared to control DLD-1 cells. **(C)** WB displayed increased β-Catenin protein expression in cells co-expressed with FSTL3. FSTL3 and β-Catenin expression is linked to the expression of EMT-related proteins in DLD-1cells. **(D)** Immunofluorescence showing that enforced FSTL3 expression increased F-actin levels. β-Catenin depletion decreased F-actin expression in DLD-1 cells, compared to control. **(E)** qRT-PCR displayed the impact of knock-down FSTL3 on β-Catenin and EMT-related genes in HCT-116 cells. ***p < 0.001; **p < 0.01; *p < 0.05. **(F)** Immunofluorescence of ectopically knock-down FSTL3 inhibited expression and nuclear translocation of β-Catenin, compared to control HCT-116 cells. **(G)** WB displayed FSTL3 expression is linked to the expression of β-Catenin and EMT-related proteins in HCT-116 cells. **(H)** Immunofluorescence showing that FSTL3 depletion decreased F-actin expression in HCT-116 cells, compared to control.

The nuclear translocation of β-Catenin is a hallmark of activated Wnt signaling and cytoplasmic β-Catenin protein levels are tightly controlled by a “destruction complex” and the 26S proteasome. Because nuclear translocation of YAP1 is modulated by the Wnt/β-Catenin pathway in melanoma-associated fibroblasts (Liu et al., 2019) and YAP1 is necessary to transactivate FSTL3 gene expression (Figure 3F), we hypothesized that elevated FSTL3 expression modulates β-Catenin signaling in CRC. The latter was supported by the fact, that abundant FSTL3 expression is obviously related to the expression of EMT-related genes, including *E*-cadherin (CDH1), *N*-cadherin (CDH2), Fibronectin-1 (FN-1), and Vimentin (VIM) in CRC tissues, illustrated by the GEPIA database (see footnote 2) (Supplementary Figure 3). To investigate any possible influence of FSTL3 on β-Catenin, we initially analyzed β-Catenin expression levels in FSTL3 overexpressed DLD-1 cells and found, surprisingly, increased β-Catenin mRNA and protein levels, compared to control cells (Figures 4A,C).

Moreover, overexpressed FSTL3 substantially increased nuclear translocation of β-Catenin, suggesting that the β-Catenin signaling pathway was activated (Figure 4B). Elevated FSTL3 expression was also linked to increased levels of F-actin polymerization which is well known to play a crucial role in EMT processes of CRC cells. The RNAi-mediated targeting of β-Catenin in FSTL3 overexpressed DLD-1 cells reduced F-actin polymerization (Figure 4D). In particular, FSTL3-overexpressed DLD-1 cells extend their antennae-like pseudopodia, which was reversible by targeting β-Catenin using RNAi. Furthermore, the qRT-PCR results demonstrated that FSTL3 overexpression was also significantly linked with diminished expression of *E*-cadherin and increased expression levels of N-Cadherin, FN-1 and Vimentin, compared to those in the control groups, which was counteracted by the β-Catenin knockdown (Figure 4A). The WB assays displayed consistent results except for FN1, which was diminished in sh-β-Catenin cells but not upregulated in cells with FSTL3 overexpression (Figure 4C). Therefore, HCT-116 sh-FSTL3 cells were utilized to further confirm the relation between FSTL3 and EMT. The qRT-PCR and WB results illustrated enhanced expression of *E*-cadherin and diminished expression levels of β-Catenin, *N*-Cadherin, and Vimentin in the HCT-116 sh-FSTL3 cells compared to the control groups (Figures 4E,G). In particular, knock-down of FSTL3, as a more reliable evidence compared to overexpression system, reduced the mRNA and protein level of FN1 (Figures 4E,G). Additionally, knock-down of FSTL3 substantially decreased the nuclear translocation of β-Catenin (Figure 4F) and reduced F-actin polymerization (Figure 4H). Altogether, FSTL3 can activate the β-Catenin pathway to promote EMT-processes in CRC cells.

### FSTL3 Regulates Glycolysis via Wnt/β-Catenin Pathway

**FIGURE 5:**
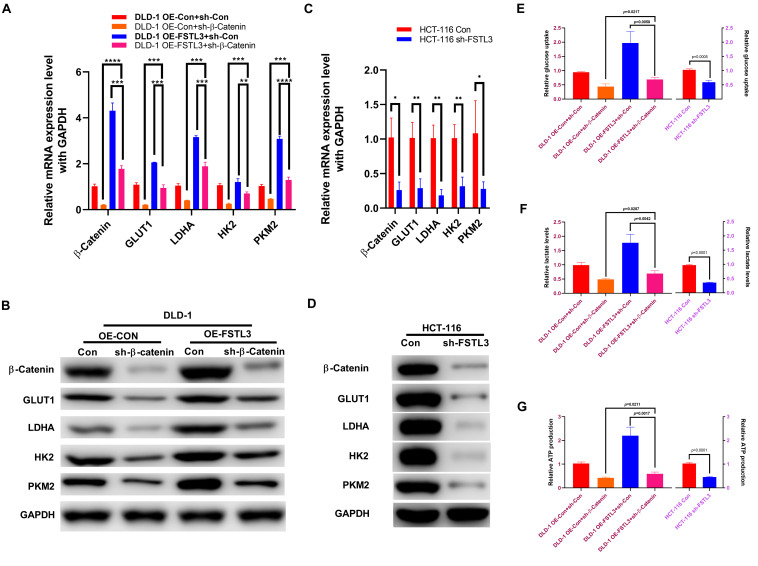
FSTL3 induces β-Catenin-mediated glycolysis. **(A,B)** qRT-PCR and WB assays displayed increased mRNA and protein expression of GLUT1, LDHA, HK2 and PKM2 in FSTL3 overexpressed and/or sh-β-Catenin DLD-1 cells, compared to control. ****p < 0.0001; ***p < 0.001; **p < 0.01. **(C,D)** qRT-PCR and WB assays displayed decreased mRNA and protein expression of GLUT1, LDHA, HK2 and PKM2 in HCT-116 sh-FSTL3 cells, compared to control. **p < 0.01; *p < 0.05. **(E–G)** Expression of FSTL3 and β-Catenin were significantly connected with glucose uptake, lactate levels and ATP production in DLD-1 cells, compared to control; knock-down FSTL3 significantly reduced glucose uptake, lactate levels and ATP production in HCT-116 cells, compared to control.

The GEPIA database (see footnote 2) confirmed that the β-Catenin expression was positively associated with genes related to aerobic glycolysis, including glucose transporter 1 (GLUT1/SLC2A1), lactate dehydrogenase A (LDHA), hexokinase 2 (HK2), and pyruvate kinase isoform 2 (PKM2) (Supplementary Figure 4). Meanwhile, the expression of FSTL3 was positively related to GLUT1, LDHA and PKM, but negatively related to HK2 expression (Supplementary Figure 5). Therefore, we wondered whether aerobic glycolysis is also involved in the phenotypic changes regulated by FSTL3 expression in CRC cells. The qRT-PCR and WB assays demonstrated that FSTL3 overexpression significantly elevated the levels of GLUT1, LDHA, HK2, and PKM2, compared to the control, while the β-Catenin knockdown was sufficient to decrease the expression levels of GLUT1, LDHA, HK2 and PKM2 in DLD-1 cells (Figures 5A,B). HCT-116 cells depleted for FSTL3 obviously decreased mRNA and protein expression of GLUT1, LDHA, HK2, and PKM2 (Figures 5C,D). In order to analyze any possible changes related to aerobic glycolysis, we investigated intracellular ATP production, lactate levels as well as glucose uptake in FSTL3 overexpressed and knockdown cells. The abundant FSTL3 expression levels significantly elevated glucose uptake and lactate levels, and promoted ATP production in CRC cells (Figures 5E–G). Targeting β-Catenin expression significantly reduced aerobic glycolysis in FSTL3 overexpressed cells, compared to the control. Altogether, FSTL3 is able to regulate aerobic glycolysis via Wnt/β-Catenin pathway in CRC cells.

### FSTL3 Promotes CRC Metastasis *in vivo*

**FIGURE 6:**
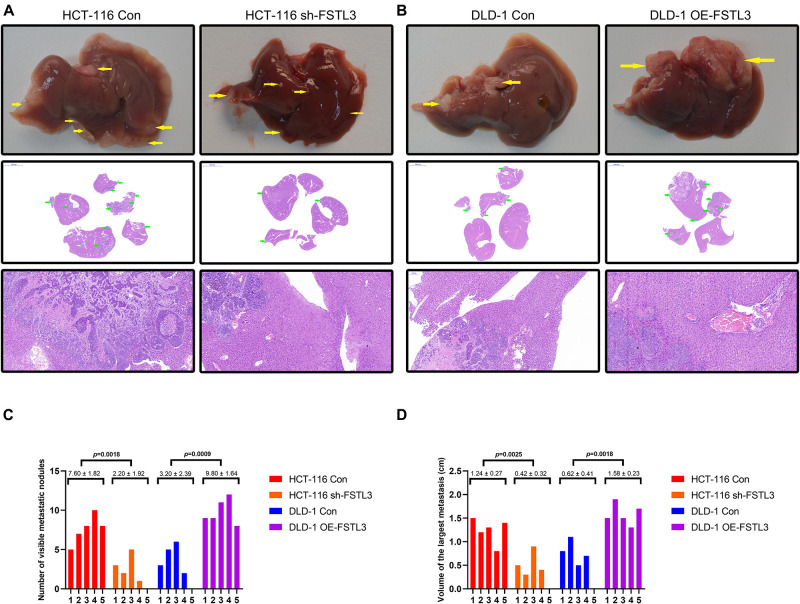
FSTL3 enhances CRC cell metastasis *in vivo*. **(A)** Liver metastases became visible in all of the mice injected with HCT-116 control cells, and in 4 of 5 mice injected with FSTL3-depleted HCT-116 cells (scale bar, top: liver specimen; middle: 5000 μm; bottom: 100 μm). **(B)** Liver metastases were visible in all of the mice injected with DLD-1 overexpressed FSTL3 (OE-FSTL3) cells, and in 4 out of 5 mice injected with DLD-1 control cells (DLD-1 Con) (scale bar, top: liver specimen; middle: 5000 μm; bottom: 100 μm). **(C)** The numbers of hepatic metastatic nodules were notably reduced in mice injected with HCT-116 sh-FSTL3 cells and DLD-1 control cells, compared with the numbers in those injected with HCT-116 control cells and DLD-1 OE-FSTL3 cells (*p* = 0.0018; *p* = 0.0009). **(D)** Volumes of the largest liver metastasis in mice injected with FSTL3-depleted HCT116 cells were significantly smaller than that with FSTL3 overexpressed cells (*p* = 0.0025; *p* = 0.0018).

In order to investigate the effect of FSTL3 on liver metastasis *in vivo*, CRC cells (HCT-116 Con and HCT-116 shFSTL3, DLD-1 Con and DLD-1 OE-FSTL3) were injected into the distal tip of the spleen using a Hamilton syringe (*n* = 5 per group). To analyze and compare any differences between FSTL3 overexpressed and knockdown CRC cells, we quantify the visible metastatic liver nodules and measured the volume of the largest liver metastasis. All of the mice successfully formed tumors in the spleen and all of the mice injected with HCT-116 control cells formed liver metastases (Figure 6A). The latter was linked to increased visible nodules and larger metastatic volumes (Figures 6C,D). In contrast, the injection of FSTL3 knockdown HCT-116 cells not only decreased the rate of liver metastasis but was also linked with significantly reduced volume of the metastatic nodules (Figures 6C,D). Additionally, liver metastases appeared in all of the mice injected with DLD-1 OE-FSTL3 cells and in 4 of 5 mice injected with DLD-1 control cells (Figure 6B). The total number of liver metastasis were obviously increased in mice injected with FSTL3 high-expression cell lines (HCT-116 Con and DLD-1 OE-FSTL3) compared to those with FSTL3 low-expression (HCT-116 shFSTL3 and DLD-1 Con) (Figure 6C). Similarly, the volume of the largest liver metastasis in mice injected with FSTL3 low-expression cells was significantly smaller than that with FSTL3 high-expression (Figure 6D). Collectively, these results suggest that FSTL3 is able to enforce the metastatic ability of CRC *in vivo*.

## Discussion

Although many advanced CRC patients can have a benefit from regional treatment approaches (e.g., surgery) for isolated liver metastases, the recurrence rate for CRC is still high, unfortunately (Choti et al., 2002; Abdalla et al., 2004; Fernandez et al., 2004; D’Angelica et al., 2011; Kawaguchi et al., 2020). In recent years it becomes increasingly apparent that the “biological footprint” of a tumor is recognized as an important and useful prognostic factor; hence the molecular profiling has undoubtedly a huge impact on risk stratification and therapy planning in CRC.

Within this study, we investigated the expression and molecular function of FSTL3 in CRC cells. Our data illustrated that abundant FSTL3 expression is a negative prognosticator in CRC patients (*n* = 130), and is significantly correlated with lymph node metastasis, staging, tumor size and IVE. The ability of cancer cells to migrate *in vivo* has a central role in cancer metastasis and it is believed that a set of specialized cells at the cancer invasive front (CIF) initiates the metastatic cascade through employing a collective mode of migration, rather than single cell migration (Yang et al., 2019). Interestingly, FSTL3 was already shown to localize to CIF, further underlying the here presented significant correlation of FSTL3 expression and lymph node metastasis in CRC. Additionally, the significant correlation of FSTL3 and IVE also reflects the intense invasive ability of cancer cells and is therefore considered as a potential predictor of metastasis.

Different molecular mechanisms have already been attributed to the development and progress of CRC and the most well-studied and dysregulated pathways belong to EGFR, Notch, PI3K/AKT as well as Wnt/β-Catenin signaling (Koveitypour et al., 2019). Wnt/β-Catenin signaling is already recognized for its ability to orchestrate various biological processes such as differentiation, organogenesis, cell proliferation and tissue regeneration. In cancer cells, Wnt is frequently found abnormally activated and accumulating evidences shows that the hyperactivation of Wnt plays an important oncogenic role, especially in CRC, and therefore representing an attractive therapeutic target for CRC treatment (Novellasdemunt et al., 2015). In fact, we found that elevated FSTL3 expression strongly promotes migration and invasion of CRC cells and that FSTL3 exerts these effects by interfering with the Wnt/β-Catenin pathway. Factors that may induce transcriptional activation of FSTL3 in CRC are unknown so far. We therefore performed FSTL3 gene promoter analysis as well as YAP1 knockout studies and found that YAP1, as a transcriptional cofactor, is required for FSTL3 transactivation. Pharmacological inhibition of YAP1 nuclear translocation drastically reduced FSTL3 expression and a positive correlation between YAP1 and FSTL3 mRNA expression was very recently shown in prostate cancer (Lee et al., 2017). Interestingly, in global gene expression analyses, YAP1 was found to transactivate genes that specifically promote cancer cell motility and consequently metastatic formation (Yang et al., 2015; Zhang et al., 2015; Lee et al., 2017; Warren et al., 2018). A possible crosstalk between the HIPPO/YAP1 and Wnt/β-Catenin signaling was already shown to play a key role in the progression course of CRC (Konsavage et al., 2012; Jiao et al., 2017). The similar biological processes mediated by the HIPPO/YAP1 and Wnt/β-Catenin signaling pathways suggest that those may cooperate in concert to regulate each other’s activity for precise regulation and fine-tuning of transcriptional target gene activation (Li N. et al., 2019). Another study reported that HIPPO/YAP1 signaling can restrict the Wnt/β-Catenin signaling by increasing Dvl phosphorylation (Varelas et al., 2010). Strikingly, YAP1 gene expression is regulated by Wnt/β-Catenin signaling in CRC cells (Konsavage et al., 2012). The cellular crosstalk between Hippo/YAP1 and Wnt/β-Catenin pathways is not fully understood, but increasing evidence have shown that both are able to coordinately regulate gene expression and signaling with relevance to cancer cell migration and metastatic formation (Azzolin et al., 2014). The importance of this crosstalk is further substantiated by the fact that overexpression of YAP1 or β-Catenin alone cannot lead to tumor development in mice, whereas co-expression of the two resulted in rapid carcinogenesis (Tao et al., 2014). Concurrent nuclear localization of YAP1 and β-Catenin appeared in most liver cancer tissues, suggesting simultaneous activation of these two pathways (Tao et al., 2014). Because of the fact that β-Catenin can induce YAP1, and YAP1 is able to transactivate FSTL3, we curiously investigated a possible impact of abundant FSTL3 on β-Catenin signaling in CRC and found not only elevated β-Catenin expression levels, but also increased β-Catenin nuclear translocation in FSTL3 overexpressed cells, suggesting a positive feedback loop. Active β-Catenin signaling depends on its nuclear translocation and is strongly linked with EMT processes and aerobic glycolysis in different cancers (Cai et al., 2018; Fang et al., 2019; Zuo et al., 2020). In CRC, dysregulated β-Catenin signaling participates in the regulation of tumor invasion, metastasis formation and aerobic metabolism, and various mutations in crucial regulatory factors of the Wnt/β-Catenin pathway have already been widely noted in CRC (Jiao et al., 2020; Wang et al., 2020; Xue et al., 2020; Zhang et al., 2020).

Our here presented data revealed that FSTL3 promotes EMT processes and enhances aerobic glycolysis by activating β-Catenin to positively affect the migrative/invasive capacity of CRC cells. In fact, EMT is able to drive a series of hybrid states, endowing cancer cells an increased metastatic and aggressive potential and is also associated with significant metabolic rewiring (Ramesh et al., 2020). Cancer cell metabolism is principally characterized by an enhanced uptake/utilization of glucose as well as lactate production. As an important intermediary in numerous metabolic processes, lactate is closely associated with tumor growth, immune escape, angiogenesis and EMT processes (Ippolito et al., 2019; Pennington et al., 2019). Especially the increased lactate production in cancer cells is thought to reduce extracellular pH, thereby promoting the acidification of the tumor microenvironment. An acidic microenvironment with less glucose available is thought to suppress immune cell infiltration and contribute to immune evasion (San-Millán and Brooks, 2017; Harmon et al., 2019). In cancer cells, the persistent activation of aerobic glycolysis can be linked to oncogene activation or loss of tumor suppressors and thereby substantially advancing cancer occurrence and progression (Jang et al., 2013). Blocking aerobic glycolysis have therefore been recently considered as a therapeutic strategy to circumvent tumorigenesis (Peng et al., 2019; Zhang et al., 2019). In line with this, we found that FSTL3 significantly elevated the levels of key metabolic enzymes like HK2, PKM2, GLUT1 and LDHA. On the other hand, FSTL3 overexpression in CRC cells that are depleted for β-Catenin, were not able to show up an increase expression of these rate-limiting metabolic enzymes, suggesting that the here presented effects largely depend on active β-Catenin. To gain more insight into any possible changes related to aerobic glycolysis and energy production, we also investigated the intracellular ATP pool, lactate levels as well as glucose uptake in FSTL3 overexpressed CRC cells. We observed that abundant FSTL3 expression was only able to increase ATP pools, lactate and glucose levels in cells positive for β-Catenin expression, whereas β-Catenin depletion circumvented any increase in the presence of FSTL3.

Finally, we analyzed the effects of FSTL3 on metastatic ability in a xenograft mouse model of CRC. We therefore injected CRC cells with showing either elevated FSTL3 (DLD-1) or depleted FSTL3 expression (HCT116) to compare any differences in visible metastatic liver nodules and to measure the largest liver metastases between both mice groups. In mice, injected with DLD-1 OE-FSTL3 cells, we observed an increase in the number of visible metastatic nodules and larger metastatic volumes, whereas injection of HCT-116 shFSTL3 cells displayed reduced nodules and a reduced metastatic volume. A very recent study came to the same conclusion, that FSTL3 is strongly linked with increased metastatic formation in a mice model of lung cancer (Gao et al., 2020). We therefore believe that abundant FSTL3 is able to enforce the metastatic ability of CRC cells *in vivo*.

However, some questions still need to be resolved in future studies. For example, dissecting the specific binding site of YAP1 on the FSTL3 gene promoter needs to be explored in more detail (e.g., ChIP). The conduction of various oncogenic signal pathways is interdependent rather than independent. This interdependence crosstalk among various signal pathways is still not fully understood and should provide new insights to uncover the relevant molecular mechanism. A dual-targeting therapy option in patients suffering from advanced disease become an attractive new strategy in recent years. Finally, the identification and better understanding of new interdependent pathways may pave the way to substantially increase a survival benefit in these difficult-to-treat late therapy stages.

## Conclusion

**FIGURE 7:**
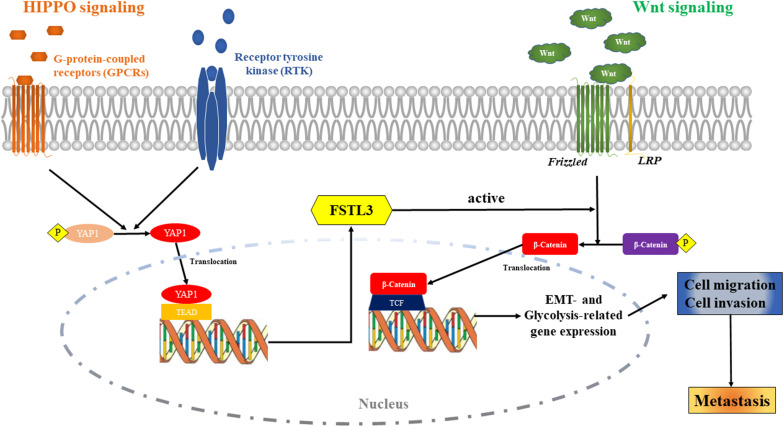
The *Cartoon*: The activation of HIPPO/YAP1 can induce FSTL3 expression, which then promotes EMT and enhanced aerobic glycolysis to affect invasion, migration and metastasis formation of CRC cells by activating the Wnt/β-Catenin pathway. FSTL3 serves as a bridging molecule in the crosstalk between HIPPO/YAP1 and Wnt/β-Catenin pathways and is a crucial regulatory factor of the molecular mechanisms governed by Wnt/β-Catenin in CRC.

The high expression of FSTL3 is a poor prognostic factor for patients with CRC. The activation of YAP1 can induce FSTL3 expression, which then promotes EMT and enhances aerobic glycolysis to affect invasion and metastasis formation of CRC cells by activating the β-Catenin pathway. FSTL3 can serve as a bridging molecule between HIPPO/YAP and Wnt/β-Catenin signaling in CRC (*cartoon*, Figure 7). Our findings illustrate that FSTL3 could serve as a crucial regulatory factor of the β-Catenin molecular mechanisms, and reflects therefore a promising therapeutic target for anti-metastatic strategies in CRC. Pharmacological targeting of YAP1 can counteract FSTL3 expression in CRC.

## Data Availability Statement

The original contributions presented in the study are included in the article/Supplementary Material, further inquiries can be directed to the corresponding author/s.

## Ethics Statement

The studies involving human participants were reviewed and approved by the Medical Ethics Committee of Xiangya Hospital, Central South University. Written informed consent for participation was not required for this study in accordance with the national legislation and the institutional requirements. The animal study was reviewed and approved by the Medical Ethics Committee of Xiangya Hospital, Central South University.

## Author Contributions

YL and FT contributed to the conception and design of the study. FT, MT, and YL performed the statistical analysis, the experimental operation, and organized the experimental data. DW, ZZ, and QP contributed to data collection and some of the experiments. YL and YH performed the statistical analysis. YL wrote the first draft of the manuscript. YL, WL, FT, and CG wrote sections of the manuscript. All the authors contributed to manuscript revision, read, and approved the submitted version.

## Conflict of Interest

The authors declare that the research was conducted in the absence of any commercial or financial relationships that could be construed as a potential conflict of interest.

## Publisher’s Note

All claims expressed in this article are solely those of the authors and do not necessarily represent those of their affiliated organizations, or those of the publisher, the editors and the reviewers. Any product that may be evaluated in this article, or claim that may be made by its manufacturer, is not guaranteed or endorsed by the publisher.

## Footnotes

1
www.clinicaltrials.gov

2
http://gepia.cancer-pku.cn/

3
http://www.proteinatlas.org

4
http://www.oncolnc.org

## Supplementary Material

Supplementary Figure 1 Vector information and partial promoter sequence of human *FSTL3* gene (NM_005860), showing three potential YAP1 binding sites (*boxes*).

Supplementary Figure 2 The data from various databases identified increased FSTL3 expression as a poor prognostic factor in CRC.

Supplementary Figure 3 Analysis in GEPIA database (http://gepia.cancer-pku.cn/) illustrated that expression of FSTL3 is obviously related to EMT-connected proteins, including CDH1, CDH2, FN1, and VIM in CRC tissues.

Supplementary Figure 4 Analysis in GEPIA database (http://gepia.cancer-pku.cn/) illustrated that β-Catenin expression is significantly associated with proteins related to aerobic glycolysis, including SLC2A1, LDHA, HK2, and PKM.

Supplementary Figure 5 Analysis in GEPIA database (http://gepia.cancer-pku.cn/) illustrated that FSTL3 expression is positively associated with expression of SLC2A1, LDHA and PKM, but negatively related to HK2.

Supplementary Table 1 The primary antibodies used in this study.

Supplementary Table 2 Primer sequences.
