# Supplementary material for: Follistatin-Like 3 Enhances Invasion and Metastasis via β-Catenin-Mediated EMT and Aerobic Glycolysis in Colorectal Cancer
Source: Front Cell Dev Biol. 2021 Jul 28;9:660159. doi: 10.3389/fcell.2021.660159 (PMC8355564; doi:10.3389/fcell.2021.660159)
Supplement: Supplementary Figure 1 — Vector information and partial promoter sequence of human FSTL3 gene (NM_005860), showing three potential YAP1 binding sites (boxes). [file Data_Sheet_1.docx]

**Supplementary Figures**


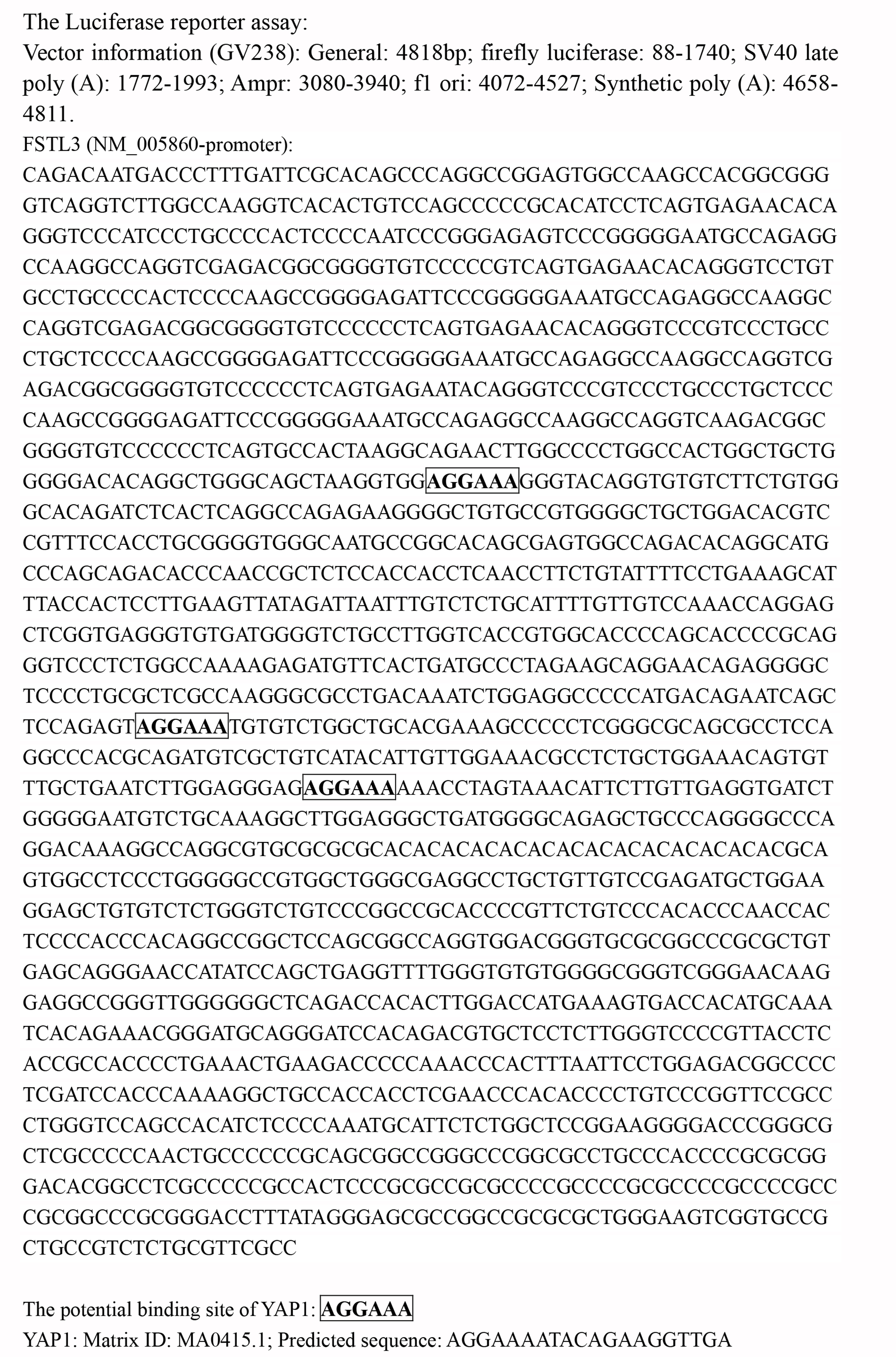


**Supplementary Figure 1:** Vector information and partial promoter sequence of human *FSTL3* gene (NM_005860), showing 3 potential YAP1 binding sites (*boxes*).


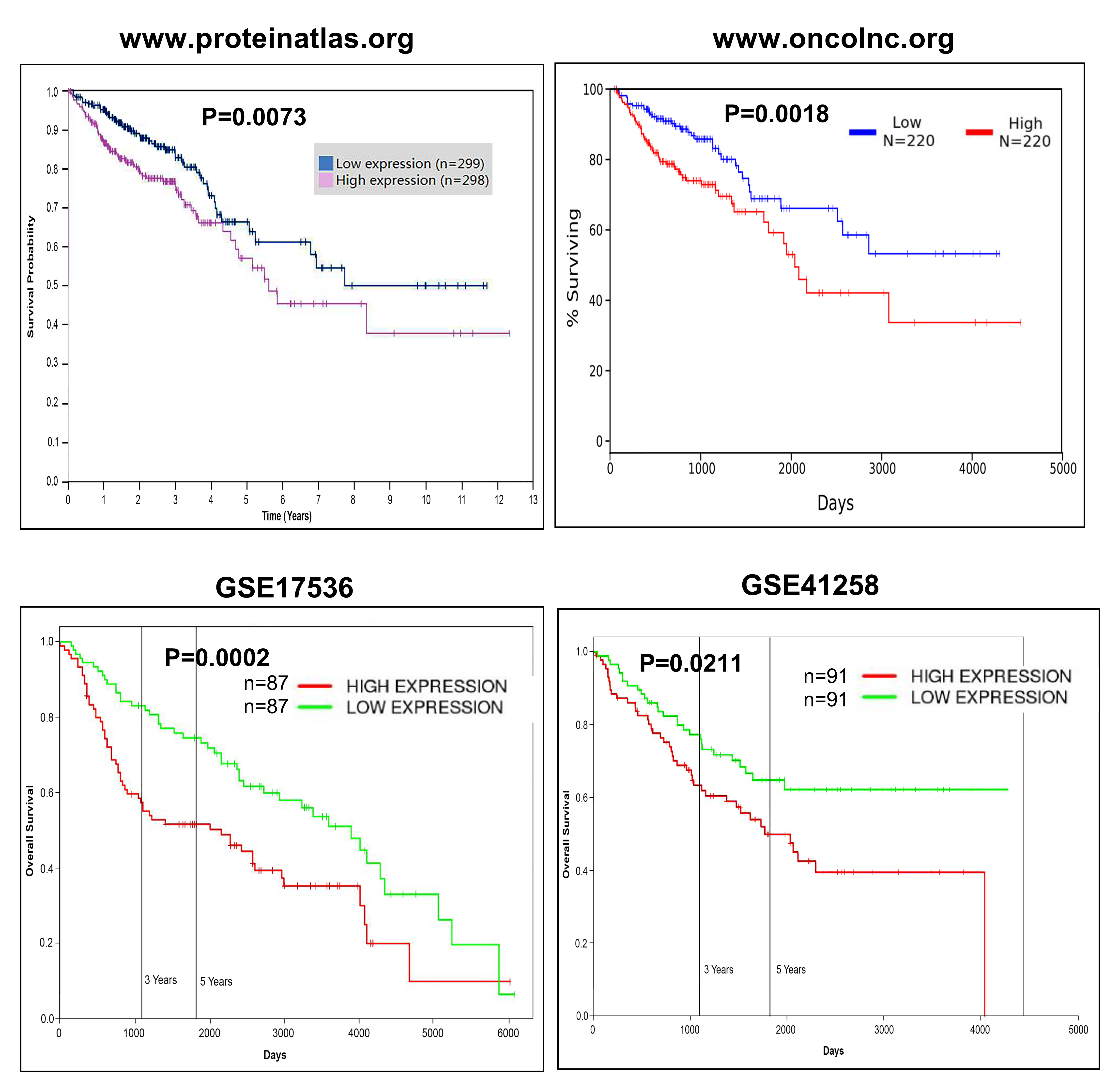


**Supplementary Figure 2:** The data from various databases identified increased FSTL3 expression as a poor prognostic factor in CRC.


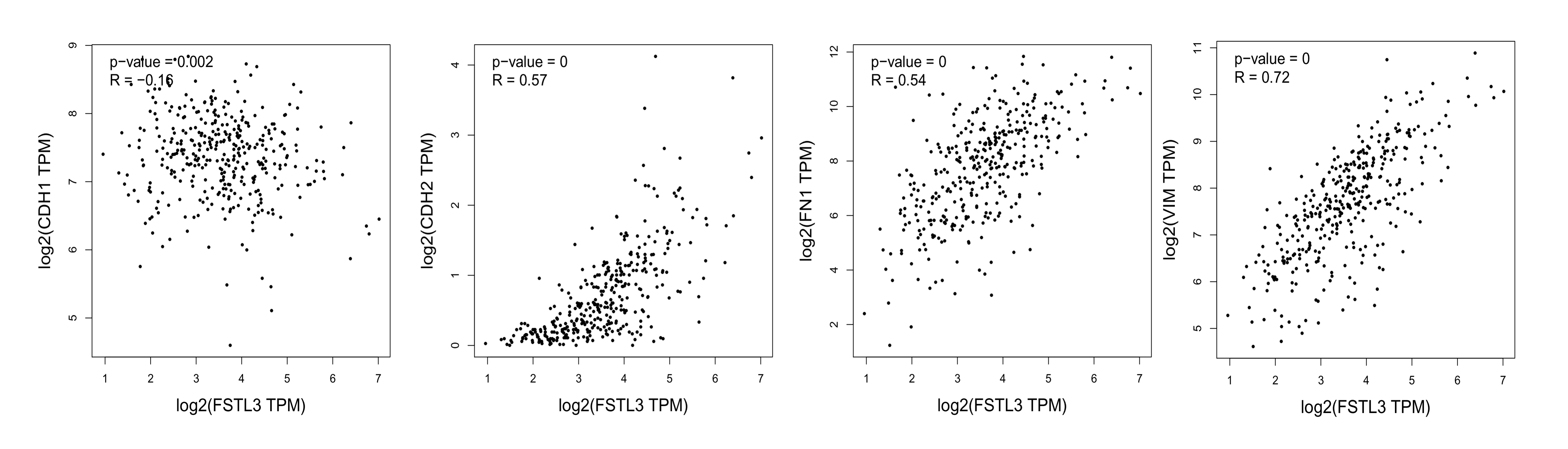


**Supplementary Figure 3:** Analysis in GEPIA database (http://gepia.cancer-pku.cn/) illustrated that expression of FSTL3 is obviously related to EMT-connected proteins, including CDH1, CDH2, FN1 and VIM in CRC tissues.


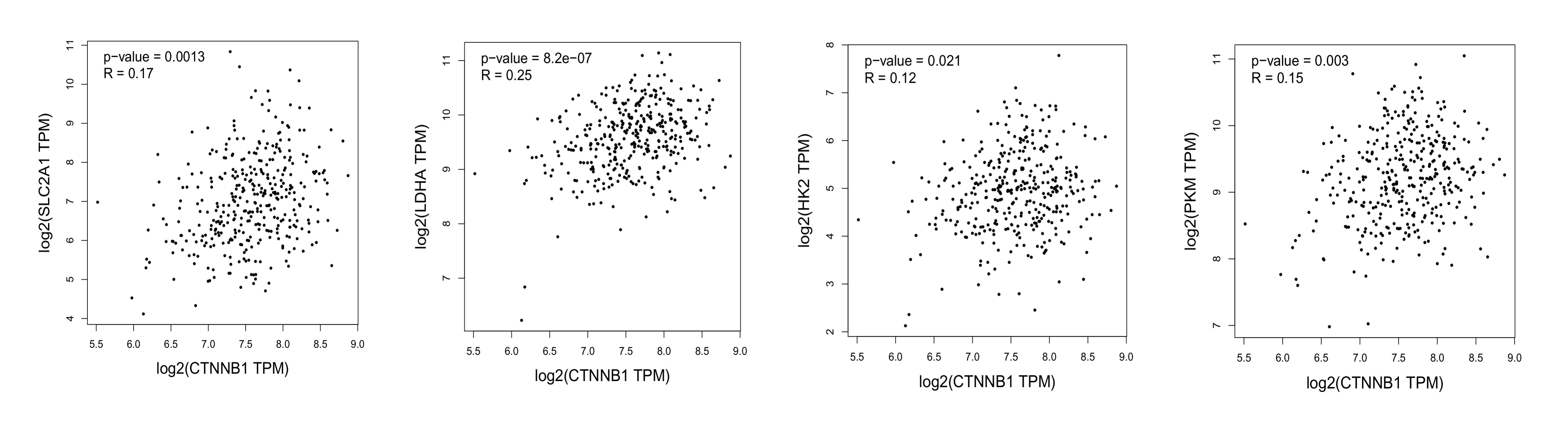


**Supplementary Figure 4:** Analysis in GEPIA database (http://gepia.cancer-pku.cn/) illustrated that β-Catenin expression is significantly associated with proteins related to aerobic glycolysis, including SLC2A1, LDHA, HK2 and PKM.


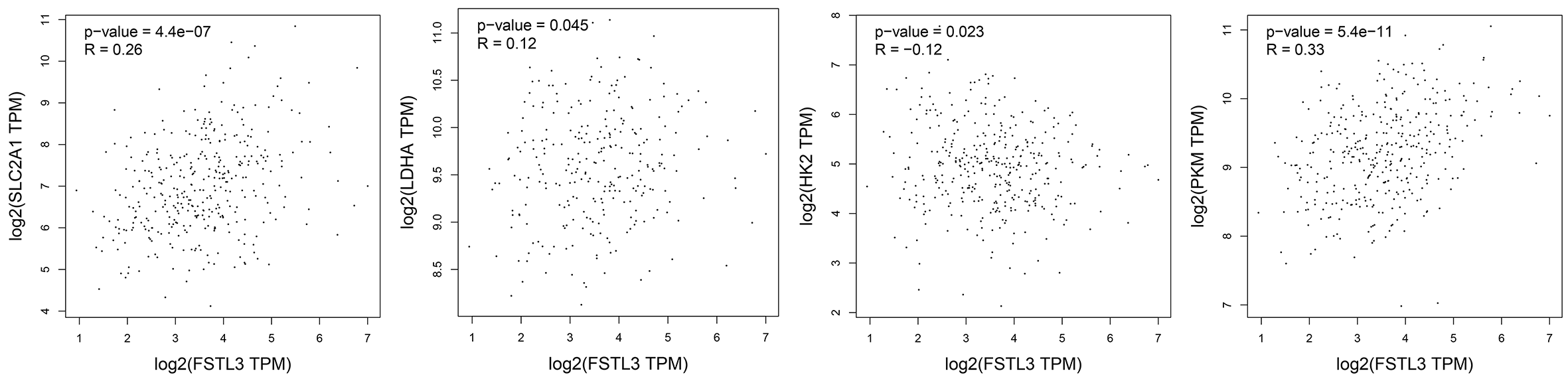


**Supplementary Figure 5:** Analysis in GEPIA database (http://gepia.cancer-pku.cn/) illustrated that FSTL3 expression is positively associated with expression of SLC2A1, LDHA and PKM, but negatively related to HK2.
