# Supplementary material for: Follistatin-Like 3 Enhances Invasion and Metastasis via β-Catenin-Mediated EMT and Aerobic Glycolysis in Colorectal Cancer
Source: Front Cell Dev Biol. 2021 Jul 28;9:660159. doi: 10.3389/fcell.2021.660159 (PMC8355564; doi:10.3389/fcell.2021.660159)
Supplement: Supplementary Table 1 — The primary antibodies used in this study. [file Data_Sheet_2.docx]

**Supplementary table 1.** The primary antibodies used in this study

| Antibody | Concentration | Specificity | Company |
| --- | --- | --- | --- |
| FSTL3 | 1:500(WB); 1:100(IHC); 1:300(IF) | Rabbit polyclonal | Abcam |
| E-cadherin | 1:1000(WB) | Rabbit monoclonal | CST |
| Vimentin | 1:1000(WB) | Rabbit monoclonal | Abcam |
| N-cadherin | 1:1000(WB) | Rabbit polyclonal | Bioss |
| Fibronectin1 | 1:1000(WB) | Rabbit polyclonal | Bioss |
| β-catenin | 1:1000(WB); 1:100(IF) | Rabbit monoclonal | Abcam |
| GAPDH | 1:4000(WB) | Rabbit polyclonal | Bioss |
| GLUT1 | 1:1000(WB) | Rabbit polyclonal | Bioss |
| HK2 | 1:1000(WB) | Rabbit polyclonal | Bioss |
| LDHA | 1:1000(WB) | Rabbit polyclonal | Bioss |
| PKM2 | 1:1000(WB) | Rabbit polyclonal | Bioss |
| YAP1 | 1:2500(WB); 1:500(IF) | Rabbit/Mouse monoclonal | Abcam |
| p-YAP1 | 1:1000(WB) | Rabbit monoclonal | Abcam |
|  |  |  |  |

**Supplementary table 2.** Primer sequences

| Gene | Forward primer (5’-3’) | Reverse primer (5’-3’) |
| --- | --- | --- |
| GAPDH | TGACTTCAACAGCGACACCCA | CACCCTGTTGCTGTAGCCAAA |
| FSTL3 | TGACACCGCCTGGTCCAACCT | CACGCCGTCGCACGAATCTTT |
| YAP1 | AGGAGAGACTGCGGTTGAAA | CCCAGGAGAAGACACTGCAT |
| PKM2 | GACTGCCTTCATTCAGACCCA | GGGTGGTGAATCAATGTCCAG |
| HK2 | CCAGTTCATTCACATCATCAG | CTTACACGAGGTCACATAGC |
| GLUT1 | ACCATTGGCTCCGGTATCG | GCTCGCTCCACCACAAACA |
| LDHA | CTGGGAGTTCACCCATTAAGCT | CAGGCACACTGGAATCTCCAT |
| β-catenin | ATTGTCCACGCTGGATTTTC | AGGTCTGAGGAGCAGCTTCA |
| Vimentin | ATTGTGGATTGTGAAGGTGA | TTCAAAAATGGTTGTGCAAT |
| E-cadherin | ATGCTATCACCTCCCCTGTGTG | CAGAAGGTTTTGTTTGAGCA |
| N-cadherin | TCAGGCGTCTGTAGAGGCTT | ATGCACATCCTTCGATAAGACTG |
| Fibronectin1 | CGGTGGCTGTCAGTCAAAG | AAACCTCGGCTTCCTCCATAA |
